# General practitioners and tutors' experiences with peer group academic detailing: a qualitative study

**DOI:** 10.1186/1471-2296-11-12

**Published:** 2010-02-12

**Authors:** Jan C Frich, Sigurd Høye, Morten Lindbæk, Jørund Straand

**Affiliations:** 1 Section for General Practice, Institute of Health and Society, University of Oslo, PO Box 1130, Blindern, N-0318 Oslo, Oslo, Norway; 2 Department of Health Management and Health Economics, Institute of Health and Society, University of Oslo, Oslo, Norway

## Abstract

**Background:**

The Prescription Peer Academic Detailing (Rx-PAD) project is an educational intervention study aiming at improving GPs' competence in pharmacotherapy. GPs in CME peer groups were randomised to receive a tailored intervention, either to support a safer prescription practice for elderly patients or to improve prescribing of antibiotics to patients with respiratory tract infections. The project was based on the principles of peer group academic detailing, incorporating individual feedback on GPs' prescription patterns. We did a study to explore GPs and tutors' experiences with peer group academic detailing, and to explore GPs' reasons for deviating from recommended prescribing practice.

**Methods:**

Data was collected through nine focus group interviews with a total of 39 GPs and 20 tutors. Transcripts from the interviews were analyzed by two researchers according to a procedure for thematic content analysis.

**Results:**

A shared understanding of the complex decision-making involved in prescribing in general practice was reported by both GPs and tutors as essential for an open discussion in the CME groups. Tutors experienced that CME groups differed regarding structure and atmosphere, and in some groups it was a challenge to run the scheme as planned. Individual feedback motivated GPs to reflect on and to improve their prescribing practice, though feedback reports could cause distress if the prescribing practice was unfavourable. Explanations for inappropriate prescriptions were lack of knowledge, factors associated with patients, the GP's background, the practice, and other health professionals or health care facilities.

**Conclusions:**

GPs and tutors experienced peer group academic detailing as a suitable method to discuss and learn more about pharmacotherapy. An important outcome for GPs was being more reflective about their prescriptions. Disclosure of inappropriate prescribing can cause distress in some doctors, and tutors must be prepared to recognise and manage such reactions.

## Background

The goal of continuous medical education (CME) is to improve the quality of health care through updating doctor's professional knowledge, skills, and attitudes. General practitioners (GPs) favour learning environments such as reading journals, discussion with colleagues, and participation in quality circles [1,2]. External audit allows doctors to review and critically analyse aspects of their clinical performance, and such feedback may include written, electronic or verbal recommendations for clinical action [3-5]. Academic detailing involves educational outreach visits and incorporates external audit and supervision, and has a larger effect on prescribing than dissemination of educational materials, audit or feedback alone [6,7]. There is little knowledge about doctors and tutors' experiences with academic detailing, and process evaluation of trials has been called for to understand how academic detailing and external audit works [4,7].

The Prescription Peer Academic Detailing (Rx-PAD) study is a Norwegian quality improvement project [8,9]. The aim of the project was to improve GPs' prescription practice for elderly patients and patients with respiratory tract infections. The Rx-PAD project was based on the principles of peer group academic detailing, and incorporated feedback on GPs' prescription patterns in addition to educational outreach visits by peer tutors in CME groups. As medical doctors with in interest in CME and quality of care, we had an interest in understanding more about how academic detailing was experienced by both GPs and tutors, in order to further improve such educational interventions.

### Objectives

We did a study to explore GPs and tutors' experiences with peer group academic detailing, and to explore GPs' reasons for deviating from recommended prescribing practice.

## Methods

### The Rx-PAD project

The Rx-PAD project was run by an academic department of general practice in collaboration with the Norwegian Medical Association, and doctors who participated received CME credits [8,9]. In Norway, GPs have to renew their clinical speciality every five years, and clinical courses and participation in a number of CME group meetings are compulsory for certification renewal. A total of 454 GPs in 80 CME peer groups were recruited and were randomised to receive a tailored intervention, either to support a safer prescription practice for elderly patients, or to improve prescribing of antibiotics to patients with respiratory tract infections. The tutors ("prescription peer academic detailers") were experienced GPs who had completed a four day educational programme about tutoring techniques and the prescription recommendations. Prescription data was collected from the GPs' electronic patient records and the Norwegian Prescription Database. The tutors met with the CME groups three times. In the first meeting tutors presented the prescription recommendations and discussed these with the group. The GPs later participated in a one-day regional workshop to learn more about the recommendations. The participants received an individual report on their baseline prescription profile. In the elderly group, GPs received a list of potential inappropriate prescriptions ("hits"), and in the antibiotic group, a prescription profile was presented. These reports were discussed at the second group meeting. New prescription data was collected one year later, a new feedback report was issued, and the groups met for a third meeting to discuss the reports.

### Participants

We considered a qualitative study, using focus groups to collect data, as a suitable method for exploring experiences with peer academic detailing among GPs and tutors who participated in the Rx-PAD project [10]. We chose a focus group design because we wanted to explore group experiences and dynamics. We conducted nine focus group interviews (FG 1-9) with a total of 59 informants, of which 39 were participating GPs and 20 were tutors (table 1). We used a strategic sampling strategy to recruit a sample of GPs that represented variation regarding prescription practice, geography, age, and gender. We approached GPs through the group coordinator and contacted GPs by phone or e-mail. Tutors were recruited at a seminar for tutors, and 10 out of a total of 13 tutors who supervised prescribing of antibiotics for respiratory tract infections and 10 out of the 14 tutors who supervised prescription patterns in elderly participated in the focus groups. We interviewed GPs and tutors in separate groups, and the rationale for our approach was that tutors as well as GPs should feel free to share their experiences with their own group, and to comment on the group processes from their point of view. All GPs and tutors in this study were participants in the Rx-PAD study, and they gave oral consent to participate in the focus groups. Participants were informed about the purpose of the present study and that anonymity would be ensured when results were presented. The Rx-PAD study has been approved by The Regional Committee for Research Ethics and The Norwegian Social Science Data Services.

**Table 1:** Characteristics of general practitioners and tutors

Characteristic	General practitioners	Tutors
Total number	39	20
Median age (spread)	47 (34-67)	52 (34-58)
Men	21	11
Women	18	9
Elderly group	20	10
Antibiotic group	19	10

### Focus group interviews

The first author (JCF) moderated eight focus group interviews and the second author (SH) was moderator for one group. The moderator used a thematic interview guide that covered experiences and views concerning the project, the group processes, and experiences with implementing changes in prescription patterns in clinical work. Specific questions that were discussed were: How did you experience getting (or giving) feedback on your prescription (participants') practice? How would you characterise the process in the peer CME group? What factors are important for your (participants') motivation for changing prescription practice? What do you think about the tutor being a GP versus other people/sources of knowledge? What do you consider as valid and relevant knowledge for your (the participants') prescription practice? What problems may inappropriate prescriptions cause? How does personal experience versus scientific evidence influence prescription of drugs? Can you give any examples of deliberate deviation from prescribing guidelines? Can you give any examples of difficult decisions concerning prescriptions that GPs encounter? How can patients' preferences influence prescription practice? How do patients get involved in or influence decisions about prescriptions?

All focus groups were recorded digitally. A preliminary analysis of each focus group was conducted shortly after the interview was done, and new themes that emerged were fed back into later focus groups for discussion. The focus groups with tutors were conducted shortly after they had tutored their CME groups for the second time. The tutors contributed with additional comments that were written down by JCF in a plenary meeting for all tutors at the end of the project (after the third CME meeting). One focus group in the elderly arm and one focus group in the antibiotics arm were conducted after the second CME group meeting, and the remaining five focus groups were conducted after the CME groups had completed their third meeting.

### Analysis

The audio-files were transcribed in verbatim by the first author (JCF) and the second author (SH). The analysis followed a procedure for thematic content analysis [11]: (i) reading all the material to obtain an overall impression and bracketing previous preconceptions; (ii) identifying units of meaning, representing different aspects of the theme and coding for these; (iii) condensing and summarizing the contents of each of the coded groups; and (iv), to generalize descriptions and concepts about the specific theme. JCF and SH analyzed the data separately and discussed categories and their contents.

## Results

Themes that emerged from the focus groups were perceived learning effects, the tutor's background and role, the peer CME groups' structure and atmosphere, the experience of receiving and discussing feedback reports, explanations for an unfavourable prescribing profile. We elaborate further on these categories below.

### GPs' perceived learning effects

Peer group academic detailing was experienced as a suitable method to learn more about pharmacotherapy, though there were participants who argued that the scheme was time-consuming. The participating GPs experienced the CME group meetings as an important arena for learning. They reported picking up good advice from others and learning practical alternatives to drugs that should not be used. GPs said their prescription data would not mirror all learning effects:

*The whole point is to reflect more, that you think twice, and with respect to this it has been a good project. It should have been done within other areas too. (GP, FG 3)*.

One important outcome for the GPs was an experience of being more reflective in decision-making about prescriptions.

### The tutor's background and role

Tutors did not consider themselves as "experts" but as "one of them". Being open about their background as GPs was an agreed upon strategy, and tutors deliberately tried to avoid being perceived as experts:

*When I presented the [quality] indicators, I said: "The next one actually surprised me a lot ... I didn't know about this before", and then they understood that I didn't think I was clever than them in the first place (Tutor, FG 2)*.

The tutors experienced that their own background was important for GPs' trust and acceptance of the project:

*It was very important ... if the Directorate for Health had sent the inspectors along with the feedback reports, then I think it would have been much more difficult ... When it comes down to the touchy parts, I think it's crucial that we're on equal terms, that we're in the same boat. (Tutor, FG 2)*.

GPs confirmed that it was important for them that the tutors were "one of them" and were independent both of the pharmaceutical industry and the health authorities. In their view, the tutors shared an understanding of the complex decision-making involved in prescribing in general practice. Guidelines could give rise to dilemmas when they considered all aspects of the patient's medical condition and situation, and thus it was easier for them to disclose reasons for inappropriate prescribing to a "GP colleague":

*We know that as a GP you have experienced these problems, and know exactly how we feel ... to disclose yourself ... and who knows how difficult it can be. (GP, FG 8)*.

Both GPs and tutors experienced that sharing the experience of being a GP contributed to an open and constructive discussion.

### Group structure and group atmosphere

Tutors reported that CME groups varied in terms of structure and atmosphere. Some groups knew little about the project, but tutors reported that there was a constructive atmosphere in most groups. Groups could still have very different "cultures", and a group with a rigid structure could be a challenge for a tutor if formal and informal group leaders insisted on running the group meetings in the usual way. Tutors introduced a new agenda and structure in existing CME groups and experienced that they had to be very explicit about the scheme:

*The groups were different ... we thought that a group is a group and all we have to do is to run the scheme ... and then I experienced that groups have their own cultures. These groups have existed for a while, which we probably have to consider in a programme like this. (Tutor, FG 2)*.

Tutors experienced lack of structure and scepticism as a challenge in some of the groups:

*At a private clinic, I had one group which was unstructured, with sceptics. I felt a great deal of "industrial adherence" rather than a critical attitude towards one's own prescription practice (Tutor, FG 2)*.

Both tutors and GPs emphasised that a "good atmosphere" in the group, and "a sense of security" among group members was essential for an open and constructive discussion.

### Receiving and discussing feedback reports

GPs' "hits" for inappropriate prescriptions in the elderly, or an unfavourable antibiotic prescription profile, was the starting point for group discussions at the second meeting. Tutors reported that GPs' tried to justify and explain their practice:

*They tried to justify their prescription practices, but I also experienced this as reflections about why they had ended up in a particular situation. (Tutor, FG 1)*.

GPs said that the feedback on their prescription profile motivated them for reflection, learning, and change. The majority of GPs shared their prescription profile in a straight-forward fashion with the other members of their CME group:

*I was surprised to see how willing people were to reflect on their own behaviour and practice ... and constantly comment like: "Well, did I really do that? I surely have to pull myself together". Very strong will, apparently, to make changes. (Tutor, FG 2)*.

GPs generally experienced the CME group as a safe setting to present and discuss their feedback reports:

*It would have been more embarrassing if it had been in a large lecture hall or a large seminar. I am not the kind of person who would present all sorts of data ... but to discuss it with three to four persons ... that's okay. (GP, FG 5)*.

Some GPs experienced disclosing their prescription profile or "hits" as frightening. Observations both from tutors and GPs indicated that discussing feedback reports had caused distress for some GPs:

*The older ones were silent, because they had a prescription profile that was far from what was recommended. The young ones dominated the discussion, and they were much more familiar with the guidelines in the first place. My impression was that the old [GPs] felt distress when disclosing their profiles ... The old ones have repeated their errors for several decades ... old habits are difficult to change. (Tutor, FG 1)*.

Tutors experienced that some GPs told that they had forgotten to bring their reports, and the tutors suspected that this was an intentional to avoid disclosing their profile or "hits":

*The ones that were afraid, didn't bring [the feedback reports] ... there was one who had failed in making any changes, and I think that was why she didn't want to present her profile. (Tutor, FG 1)*.

GPs were generally more embarrassed if they had hits they knew they should have avoided, such as prescribing flunitrazepam to elderly patients, compared to potentially harmful drug combinations that had not been highlighted in the recommendations.

### Patients' needs and demands

Patients' demands for certain drugs were commonly used as explanation for an unfavourable prescription profile. GPs explained that they had tried to stop prescribing certain drugs after they joined the project, but had failed to convince their patients that this was a good solution for them. A typical example was a patient who had been using a benzodiazepine hypnotic for several years:

*The patient had used nitrazepam 5 milligrams for five, six, seven years, and then I learned that we should use zopiclone, which does not work, ok, then we try the other, zolpidem or something like that, and he is not happy and demands nitrazepam. And then he gets nitrazepam and he sleeps well. If you are born in 1919 and are 87 years old, then I don't see the reason why we should change this. (GP, FG 6)*.

GPs feared that changing to another drug in elderly patients could cause distress to certain patients:

*It is important that you know your patients well. You may make it worse by withdrawing a drug if they are nervous. (GP, FG 8)*.

The GPs generally said that it was easier not to prescribe an inappropriate drug in the first place, than to withdraw a drug from a patient. The taste of the drug was a common explanation for not prescribing the recommended first-line drug for respiratory tract infections in children:

*One issue when it comes to children is taste [of the antibiotic] ... we have to give them something they [will] take, and then you find a favourite that works well. (GP, FG 3)*.

Generally, GPs found it easier not to prescribe an inappropriate drug in the first place, than to withdraw a drug a patient had used for some time. GPs in the antibiotic group said there had been an increase in atypical pneumonias the winter the project started, and that this could have influenced their antibiotic prescriptions.

### The GP's background and the practice

A common explanation for "hits" was the patient was not really their patient, because one had just issued the prescription on behalf of a colleague who was absent. The notion of "summer holidays prescriptions" denoted this phenomena. If the patient's regular GP was absent, the receptionist would ask one of the other GPs to issue the prescription that the patient needed. As long as the drug was one the patient's list of drugs in the electronic patient record, one rarely questioned the indication for the drug:

*When we work in a large health centre, then we sign prescriptions for each other ... when a colleague is absent, we issue prescriptions for him that day. Any prescription I issue is my responsibility, but if you are asked to prescribe a particular drug [for a colleague] then you sign it in the reception. I don't check which other drugs that person uses. (GP, FG 6)*.

A usual explanation for issuing inappropriate combination of drugs was that the combination had been "imported", for example that they had been prescribed by the patient's previous regular GP. In this situation, one was reluctant to question problematic drugs or combination of drugs before trust had been established in the doctor-patient-relationship. Other explanations were that it could be difficult to know the complete list of all drugs a patient used. GPs explained that also a local "prescription culture" could influence patients' expectations, which in turn could influence GPs' prescription habits.

### Other health professionals or health care institutions

Other health care institutions, such as nursing homes, community nursing services, or hospital specialists could have a role in inappropriate prescribing:

*How detailed should we check what the community nurses order? My secretary print it and I sign it, and three drugs are listed. It could be a fourth drug the patient uses that I don't know about, right. That was quite unpleasant. (GP, FG 6)*.

GPs said that hospital specialists influenced their prescribing practice, because the specialists prescribed new treatments without considering whether the new drug was appropriate in relation to the patient's other medication:

*Very much is about tradition, and we are influenced by the feedback from other colleagues, especially from hospital. Some of my tetracycline [prescriptions] are prescribed for elderly patients and patients with chronic obstructive lung disease who have exacerbations that the hospital treat with tetracycline ... Obviously this influences us. (GP, FG 4)*.

A typical example was patients with chronic obstructive pulmonary disease where a hospital specialist both had prescribed the treatment and provided written instructions about this to the GP in the discharge report.

## Discussion

### Principal findings

A shared understanding of the complex decision-making involved in prescribing in general practice was reported by both GPs and tutors as essential for an open discussion in the CME groups. Tutors experienced that CME groups differed regarding structure and atmosphere, and in some groups it was a challenge to run the scheme as planned. Individual feedback motivated GPs to reflect on and to improve their prescribing practice, though feedback reports could cause distress if the prescribing practice was unfavourable. Explanations for inappropriate prescriptions were with lack of knowledge, factors associated with patients, the GP's background, the practice, and other health professionals or health care facilities.

### Methodological considerations

This study explores GPs and tutors' experiences with being participants in a peer academic detailing project. We designed the study and approached the data with a specific interest, and may have overlooked phenomena that would otherwise have been described in a study with a broader aim. Focus group interviews are suitable for generating data on group meanings and processes. Such interviews provide indirect information about the group processes, and should not be considered as observational data about what went on in the groups during the project. We have triangulated data about the group process from GPs and tutors, which has made our analysis and findings robust. We think data from the focus groups are valid for participants' experiences with the project, and we consider our findings valid and transferable to quality improvement work among experienced GPs in peer CME-groups. Individual interviews would probably have been more suitable for a further exploration of sensitive issues and experiences of distress among participants.

### Disclosure of performance in quality improvement work

Lack of structure and resistance in the group, and reluctance to disclose ones' profile, may represent general challenges for peer CME groups, but may also reflect group members' strategies to cope with feelings of distress and unease. Our findings suggest that disclosure of inappropriate prescribing can cause distress in GPs, though the majority seemed to be comfortable with discussing their reports with peers in the CME group. Our results are in concordance with research that suggests that GPs may feel disappointment if their prescribing practice conflict with their ideals [12]. The findings underscore that tutors have an important role in managing distress and contributing to an informal and relaxed atmosphere in peer academic detailing groups. Further research should investigate how different formats for feedback, such as individual counselling versus discussions in peer groups, are experienced by participants in quality improvement projects. It would also be interesting to study how such factors related to the process may impact on the outcome of quality improvement interventions.

### Drugs and decisions in context

Inappropriate prescriptions and deviations from guidelines can be deliberate or unintentional. We found that GPs explained inappropriate prescriptions by factors linked to the patient, the GP and the practice, other health professionals, or health care institutions. These results suggest that inappropriate prescribing can not be explained by lack of knowledge only. In accordance with previous research, we found that prescription of drugs involves complex decision-making processes [13]. Patients' beliefs about the effectiveness and beliefs about drugs may differ significantly from current medical knowledge [14,15]. Our findings suggest that patients' expectations and the doctor-patient-relationship influence GPs decisions about prescriptions. Incorporation of evidence into everyday practice may be more effective if an intervention contribute to local consensus building [16]. It could have been helpful for the participating GPs if controversial issues and local traditions had been discussed with representatives from specialist health care in an attempt to build local consensus. The health centre or practice, including GP colleagues, is another important context where prescribing behaviours and opinions are shaped. It may be easier to make changes in one's own prescribing behaviour if the whole practice is engaged and motivated to reflect on and change the "practice culture". To what extent measures to change the practice culture influence individual GPs prescribing should be subject to further study. Our data suggests that GPs give many and various reasons for inappropriate prescribing and our findings here are in line with previous research [12,17,18]. Some explanations "export" the problem to other doctors or institutions. Though many factors play a role for prescribing, many factors are within the scope of GPs' influence, and it is the tutor's task to help the group and the individual GP to recognize liability and to define realistic targets for improvements and change.

### Balancing interests and concerns

Balancing interests and concerns is an essential aspect of GPs' work [19]. Previous research suggests that GPs try to balance best practice against perceived patient satisfaction when deciding whether to prescribe antibiotics or not for patients presenting with acute respiratory infections [20]. Summerskill & Pope explored barriers to secondary prevention of coronary heart disease, and found that GPs frequently contrasted "cold" evidence with the "warm" relationship they had with patients [21]. GPs desire to preserve a good relationship and to maintain compliance with other treatment regimes sometimes was more important than implementing secondary prevention. Our study suggests that GPs balance perceived benefits and harms, and aim to make a decision that is appropriate for the patient at hand. Inappropriate prescriptions may thus sometimes be a result of a deliberate balancing of various interests and concerns. Tutors in the Rx-PAD project seem to have negotiated a role where they recognize the complexity of everyday clinical practice, while still being able to focus on the potential for quality improvement. Rather than focusing on what one should not do, a tutor should focus on the benefits when arguing for an evidence-based practice [22]. One of the important learning effects the GPs reported was becoming more reflective about their own practice, and further research should study such subjective outcomes in addition to outcomes related to actual prescribing behaviour.

## Conclusions

GPs and tutors experienced peer group academic detailing as a suitable method to discuss and learn more about pharmacotherapy. An important outcome for GPs was being more reflective about their prescriptions. Disclosure of inappropriate prescribing can cause distress in some doctors, and tutors must be prepared to recognise and manage such reactions.

## Competing interests

The study was funded by The National Board of Health and The Norwegian Medical Association. JS and ML are principle investigators for The Prescription Peer Academic Detailing (Rx-PAD) study.

## Authors' contributions

JCF was principal investigator for this study. JCF designed the study in collaboration with JS and ML. JCF and SH collected the data. JCF and SH coded and analysed the data, and JCF wrote the first draft of the manuscript. All authors have critically revised of the manuscript and participated in the interpretation of findings. All authors have read and approved the final manuscript.

## Pre-publication history

The pre-publication history for this paper can be accessed here:

http://www.biomedcentral.com/1471-2296/11/12/prepub
